# Good Saves: How Emergency Medicine Residents are Learning From Success

**DOI:** 10.7759/cureus.49508

**Published:** 2023-11-27

**Authors:** Leah Bralow, Eleni McCaffery, Scott Leuchten

**Affiliations:** 1 Emergency Medicine, St. Barnabas Hospital Health System, New York, USA; 2 Emergency Medicine, Sophie Davis School of Medicine, City University of New York, New York, USA

**Keywords:** survey research, positive psychology, recognition, case-based teaching, case based learning, innovation in medical education, resilience and well-being, morbidity and mortality, teaching in emergency medicine

## Abstract

Introduction: The practice of learning from medical errors is well-established and well-researched in the literature on morbidity and mortality conferences. However, durable learning from case-based education occurs not only through the analysis of medical errors but also through the evaluation of how critical decisions were made to result in a positive clinical outcome, what we will call a “good save.” The aim of the current study is to provide an overview of how US-based emergency medicine residencies are teaching using "good saves.”

Methods:A national survey of emergency medicine (EM) residency leadership was distributed through the Council of Residency Directors (CORD) and the Society of Academic Emergency Medicine (SAEM) listservs. A descriptive analysis of the results was undertaken.

Results: Residency leadership representing 67 different US EM training programs participated in our survey. Of these, only 19 programs use formal learning objectives and dedicated education time to teach from "good saves." Thirty-six programs provide informal recognition, often in the form of a “shout-out.” Residency leadership is motivated to provide this recognition and learning through efforts to promote wellness and resiliency among EM residents. Notably, the use of prizes and awards is not necessary.

Discussion: Some EM residencies in the United States are making targeted efforts to promote the recognition of successful clinical care. This recognition and education are being used as tools both to promote wellness and to teach resiliency. However, there is wide heterogeneity in approaches. Our survey provides examples of the many ways that “good saves” can be incorporated into any EM residency curriculum with the potential for significant impact.

## Introduction

The practice of learning from errors is well established and the inclusion of morbidity and mortality conferences (M&M) in residency training has been an Accreditation Council for Graduate Medical Education (ACGME) requirement since 1983 [1]. Despite this longstanding mandate, the M&M format is highly variable both between specialties and within the specialty of emergency medicine (EM) [2,3]. There are many published efforts to avoid feelings of persecution or guilt during M&Ms. Often, these efforts meet with only moderate success [3,4]. Regardless, residents who take ownership of mistakes and discuss them in a formalized setting report durable changes in clinical practice [4]. This creates a double-edged sword effect for M&Ms. Programs need to walk a fine line to promote learning from mistakes without creating a culture of “shame and blame” [2,5,6].

In a study conducted by learning psychologists Eskreis-Winkler and Fishbach [7], participants were shown to learn the most from personal success. Learning achieved by observing others’ successes and learning achieved by observing others’ failures were both found to have medium efficacy and to be statistically equivalent. Finally, participants learned the least from personal failures. The authors hypothesize that individuals learn the least from personal failures because of a perceived threat to ego, which causes the learners to tune out and stop processing information. While the Eskreis-Winkler study provides a basis for learning reinforcement through success, medical literature regarding intentional learning from positive outcomes is lacking.

In recent years, high-risk professional fields such as aviation and air traffic management have undergone a significant shift in learning. These industries have added a focus on frequent assessments of when a team successfully manages a complicated situation resulting in a good outcome [6,8]. Similar to aviation and air traffic management, healthcare teams that overcome challenges in diagnostics or care exemplify resilient performance [6]. These instances where decisions are nuanced and challenging are fraught with potential for failure which could result in harm to the patient. Residencies in all specialties of medicine have an opportunity to teach from case-based evaluation of how critical decisions were made to result in a positive clinical outcome. We call these cases "good saves." "Good saves" are cases in which the team successfully navigated complex and nuanced decisions to reach a successful clinical outcome.

Medicine is in the early stages of incorporating reinforced learning from success. Many hospitals now have staff recognition awards such as “nurse of the month” or “physician of the year” awards. While important for wellness, these awards fail to teach trainees how to become exemplary clinicians. Learning from success has been shown on a small scale to be a powerful tool in medical quality improvement initiatives [9]. Formalized advice on how best to learn from successes in medicine is lacking.

Our department is interested in providing both recognition and education using case-based learning to analyze critical decision-making involved in the successful clinical care of complicated cases, i.e., by using “good saves”. To learn how to best incorporate “good saves” into our EM residency curriculum, we conducted a national survey of EM residency program leadership. The aim of the current study is to provide an overview of current strategies among US EM residencies to educate residents using “good saves.” We hypothesized that there would be a large amount of heterogeneity in how this education is incorporated into EM residency training, similar to published literature on M&Ms [2,3,5].

## Materials and methods

We conducted a survey of US EM residency leadership to determine if the programs they represent provide recognition of or learning from “good saves” and in what format this education is provided. One response was requested per program. At the time of survey distribution, there were 262 ACGME-recognized EM residencies in the United States. Participation by program leadership was voluntary.

The survey instrument was divided into three sections: (1) programs that do not provide any form of recognition or education; (2) those that provide informal recognition; and (3) those that provide formal education on “good saves.” The survey was administered using a web-based survey tool (Google Forms, Mountain View, CA). Responses were collected anonymously, and the zip code was used to identify and remove duplicate responses so that each program would be represented only once (in the case of one zip code that has two programs in it, additional demographics such as program size and number of residents per PGY-year were used to differentiate responses from the two different programs). If a respondent did not answer all questions, their complete answers were included in the analysis. 

The survey instrument was distributed via email to the leadership of each EM residency listed in the SAEM Directory between November 2021 and February 2022. The survey was distributed using the Council of Emergency Medicine Residency Directors (CORD) listserv, the SAEM Education Section listserv, and individually via email to the contact listed for each program on the SAEM Residency Directory [10]. The survey was to be completed by a member of residency leadership with in-depth knowledge of the residency’s curriculum. Non-responders received two follow-up emails which were sent to the program contact listed in the SAEM Residency Directory.

We defined formalized education based upon “good saves” to be the assignment of learning objectives to the case review and distribution of the educational content to the residency in either a verbal or written format. Informal recognition was defined as an acknowledgment of successful clinical care in either a formal verbal or written format without the assignment of learning objectives. Formal verbal recognition was considered to be recognition provided at either a departmental or residency-wide meeting or during some sort of education conference.

Data was analyzed using descriptive statistics to evaluate the state of learning from "good saves" amongst residencies in our survey sample.

## Results

Responses were received from the representatives of 67 unique ACGME-recognized US EM residency programs (Table 1). Around 70% of respondents were members of EM Residency program leadership as defined by being either a program director or an associate/assistant program director. The remaining 30% were other faculty heavily involved in residency education such as MedEd fellows or department chairs. In total, 26 of 42 states with EM residencies are represented in our survey sample.

**Table 1:** Demographics of Respondents

Program Demographic Data	n (%)
Number of PGY-years	
3-year	49 (73.1%)
4-year	14 (22.4%)
Combined/Extended Training Program	3 (4.5%)
Number of residents per PGY-year	
Average	12.7
Range	2 – 22
Training Environment	
Academic Hospital	47 (70.1%)
Community Hospital	21 (31.3%)
County Hospital	14 (20.9%)
Critical Access Hospital	1 (1.5%)
Location	
Northeast	23 (34.3%)
Southeast	10 (14.9%)
Midwest	13 (19.4%)
Southwest	10 (14.9%)
West Coast	7 (10.4%)
Northwest	4 (5.9%)
Role of respondent	
Program Director	27 (40.9%)
Assistant/Associate Program Director	19 (28.9%)
Program Coordinator	2 (3%)
Core Faculty	7 (10.4%)
Other Faculty	12 (17.9%)

Seventy-three percent of responding programs were three-year residencies, 4.5% were extended training programs and the remainder were four-year residencies. The average number of residents per year of responding programs was 12.7 residents per PGY-year.

Of 67 programs, 82% (55 programs) reported that they participate in some form of departmental recognition of “good saves” (Table 2). The majority of these (65.4%, 36 programs) use informal recognition to provide support to their faculty and residents. The remaining 19 programs (34.5%) have instituted formalized educational processes to learn from excellent clinical care. The majority of these (15/19) utilize this educational format on a monthly basis.

**Table 2:** Recognition of Good Saves

Survey Question	n (%)
Is recognition provided?	
Yes	55 (82%)
No	12 (17.9%)
Style of recognition:	
Informal recognition (“shout out” and similar)	36 (65.4%)
Formal recognition with assigned learning objectives	19 (34.5%)
Are incentives or awards provided?	
Yes	2 (3.6%)
No	53 (96.3%)

Of the 19 programs that provide formalized education on “good saves,” all utilized a lecture format and some included an additional mechanism of recognition (Table 3). Ten programs incorporate “good saves” as part of a M&M conference. The remaining programs utilize a stand-alone lecture unaffiliated with the M&M conference. The presenter for these educational conferences is most commonly program leadership (68%) followed by a chief resident or core faculty. Some programs (n=9) also reported using a second form of recognition - for example, an email, small group didactic, or blog post in addition to the lecture.

**Table 3:** How Departments Accomplish Formal Education of Good Saves

Survey Question	n (%)
How are cases identified within your department?	
Self-report to an email address	15 (78.9%)
Word-of-mouth	14 (73.7%)
Chart extraction	5 (26.1%)
Administrative identification	8 (42.1%)
Who is responsible for presenting "good saves" education?	
Department administration	3 (15.8%)
Residency leadership faculty	13 (68.4%)
Core faculty	8 (42.1%)
Faculty	4 (21.1%)
Chief residents	10 (52.6%)
Other residents	7 (36.8%)
When are "good saves" taught?	
Asynchronous to conference	4 (21.1%)
During weekly didactics (large group conference or small group daily teaching conference)	18 (94.7%)
During faculty meeting	4 (21.1%)
Other	1 (5.3%)
Are the patients invited to attend the discussion of their care?	
Yes	1 (5.3%
No	16 (84.2%)
Depends on the situation	2 (10.5%)
What educational format is used?	
Email	5 (26.3%)
Blog post	1 (5.3%)
Small group didactics/case presentation (eg- morning report, small group session in conference)	5 (26.3%)
As part of the M&M conference	10 (52.6%)
Standalone lecture during weekly didactics conference (ie- unaffiliated with M&M)	9 (47.4%)
Other	2 (10.5%)

**Figure 1:**
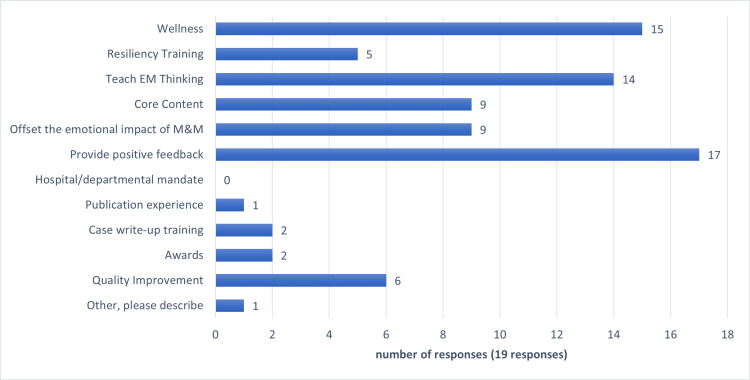
Motivators for Utilization of Good Saves Among Emergency Medicine Residencies

The formalization of “good saves” education serves many purposes for the EM residencies that utilize it (Figure 1). Program leaders report that their top three reasons for utilizing this method of teaching are: to provide positive feedback (17/19), wellness (15/19) and to teach EM thinking (14/19). Other common reasons to use “good saves” are: to provide core content material (9/19) and to offset the emotional impact of the M&M conference (9/19). As some institutions view “good saves” as a counterpoint to M&M, “good saves” are presented in many departments at the same frequency as M&M.

Cases tend to be self-reported by the physicians involved via email or come to the attention of program leadership through word-of-mouth. Overwhelmingly, the patients involved in these cases are not invited to attend the discussion of their medical care (84%).

Of those programs that provide formalized education, program leadership overwhelmingly feels that residents enjoy the recognition and discussion of “good saves” that occur in various forms, with 100% of responses in the positive domains of the Likert scale.

Many programs (55%) report the use of informal methods to recognize “good saves” (Table 4). These forms of recognition are highly varied with the most popular form of recognition being “other,” followed by “email” in 54% of programs. Respondents provided examples of “other” forms of recognition which thematically center around providing some sort of certificate of recognition or announcement to the department. Announcements were made during department meetings, sometimes as part of M&M, via email, or as a “shout out” on a group messaging platform.

**Table 4:** Informal Recognition of Good Saves

Survey Question	n (%)
Means of recognition	
Email	20 (54.1%)
Recognition board	7 (18.9%)
Group Text message	5 (13.5%)
Note (paper, e-card, etc)	2 (5.4%)
Other: Verbal announcement at a department activity	19 (51.3%)
Other: Newsletter	2 (5.4%)
Frequency of recognition	
Daily	2 (5.4%)
Weekly	10 (27%)
Bi-Weekly	0 (0%)
Monthly	19 (51.4%)
Quarterly	0 (0%)
As cases occur	12 (32.4%)
Awards and/or gifts for recipients	
Yes	2 (5.4%)
None	17 (45.9%)
No answer	18 (48.6%)

Nineteen (51.4%) programs provide informal recognition monthly. Few programs provide incentives or a reward, however, two respondents surveyed mentioned giving out a certificate of appreciation or a gift card.

Twelve respondents (18%) reported that there is no mechanism at their residency program to provide recognition or education from “good saves.”

## Discussion

Our data show that some EM residencies in the United States utilize “good saves” as an educational tool to teach clinical thinking. As expected, there is high variability between programs. Few programs are providing formalized education from “good saves.” It is currently more common for programs to utilize informal recognition modalities. Regardless of style, most of the residencies that use “good saves” provide education or recognition monthly. Most programs utilize a standardized recurring meeting or lecture to include recognition. For example, many programs that provide formal education on “good saves” tie this education into M&M conferences. 

A challenge facing programs that wish to incorporate “good saves” into their education is identifying cases. As opposed to morbidity and mortality, where hospital-based error reporting systems can be used to assist with the identification of cases for discussion, our survey population reported that “good saves” are typically identified by word of mouth. This lack of structure is not surprising. A culture of analysis of positive outcomes is not yet present in the American healthcare system. Creating a culture of recognition and an official process for submitting a case to be recognized as a “good save” can be as easy as encouraging an email to a specific faculty member or setting up an electronic submission form.

Of emergency medicine residencies that provide recognition of “good saves,” many reported that these initiatives are motivated by a desire to promote wellness and resilience amongst their physicians. Physicians in all fields of medicine are overcoming obstacles and navigating complex decision-making that could easily have led to failure in every success story. Successful cases that require physicians to overcome challenges exemplify resilient performance [6]. It is simple to convert what was informal recognition to valuable, durable learning for our residents using learning objectives. Creating appropriate learning objectives generates focus and connects what we intend to teach with the reality of what we are teaching [11, 12]. The thoughtful creation of case-based learning objectives allows educators to appropriately highlight the most important aspects of resilient clinical care shown by the case. This can benefit our trainees as a form of resiliency engineering.

Our results also show that the recognition itself provides a reward for physicians. Physical rewards such as certificates or gift cards are rarely utilized. Physicians inherently strive for perfection in clinical care. As a group, we have strong intrinsic learning motivators. Even a small amount of recognition can provide excellent reinforcement.

This study has several limitations. The primary is the small number of EM residencies who participated - only 25% of ACGME-recognized US EM residency programs at the time of the survey were represented by respondents to the survey. We obtained responses from a geographically wide area, with 26 states represented in our sample, but a larger sample size would have increased our geographic distribution and the accuracy of our representation of the state of current practices in US EM residencies. Additionally, as is the nature of survey studies with voluntary participation, programs that already provide recognition or education about “good saves” are more likely to self-select for the research survey.

## Conclusions

Our survey shows that some US EM Residencies are starting to recognize the importance of acknowledging physicians for exemplary clinical care. A small portion of residencies are assigning learning objectives to “good saves” and these programs set aside specific educational time to these cases. Residency leadership are motivated by efforts to increase wellness, resiliency and train EM thinking. Some barriers to operationalizing this may be lack of widespread knowledge of the benefits of learning from success and a lack of formalized reporting process to identify “good saves”. Our results show that reporting processes need to be developed but can be as easy as an email account. Our results also show that recognition is the reward. Physical prizes are not necessary. In the future, more programs can adopt “good saves” - in whatever format suits each residency program best. Future research can be conducted to establish resident perspectives on “good saves” and evaluate the durability of this type of learning.
